# Penile metastasis from primary cholangiocarcinoma: the first case report

**DOI:** 10.1186/1471-230X-13-149

**Published:** 2013-10-14

**Authors:** Antonio Luigi Pastore, Giovanni Palleschi, Giorgia Manfredonia, Piero Maceroni, Domenico Alvaro, Domenico De Santis, Simone Ferretti, Natale Porta, Claudio Di Cristofano, Carlo Della Rocca, Andrea Fuschi, Yazan Al Salhi, Cristina Maggioni, Davide Moschese, Vincenzo Petrozza, Antonio Carbone

**Affiliations:** 1 Department of Medico-Surgical Sciences and Biotechnologies, Urology Unit, ICOT Latina, Sapienza University of Rome, Latina, Italy; 2 CADI, Radiology Department, Sapienza University of Rome, ICOT Latina, Latina, Italy; 3 Department of Medico-Surgical Sciences and Biotechnologies, Gastroenterology Unit, ICOT Latina, Sapienza University of Rome, Latina, Italy; 4 Department of Medico-Surgical Sciences and Biotechnologies, Histology Unit, ICOT Latina, Sapienza University of Rome, Latina, Italy

**Keywords:** Cholangiocarcinoma, Elastography, Magnetic resonance, Penile metastases

## Abstract

**Background:**

Metastatic penile carcinoma derived from cholangiocarcinoma (CCA) has not been previously reported in the literature. Common metastatic sites for CCA include the regional lymph nodes and adjacent organs. CCAs are not highly vascularised tumours, making hematogenous metastases uncommon. Hematogenous CCA metastases commonly occur at distant organs such as the lungs, adrenal glands, and bones. Median survival for patients with metastatic disease is generally less than 1 year.

**Case presentation:**

A 74-year-old Caucasian man consulted us after having undergone penile ultrasonography for pain and increased thickness at the base of the penis after self-examination. The patient presented with a history of hepatitis C-related cirrhosis and intrahepatic CCA, diagnosed 3 years previously. A biopsy of the corpora cavernosa on both sides revealed a carcinoma harbouring the same histological and immunophenotypical features as the primary hepatic lesion.

**Conclusions:**

To date, there is no case of penile or urogenital system metastasis from CCA described in the literature. Therefore, this article represents the first case report of penile metastasis from CCA.

## Background

Cholangiocarcinoma (CCA) is an uncommon and aggressive malignancy that originates in the bile duct epithelium and is associated with very poor prognosis [1]. The incidence and risk factors of CCA vary in different regions of the world. The highest incidence is reported in the north-eastern part of Thailand. The major risk factor for CCA in this area is *Opisthorchis viverrini* infection, whereas *Clonorchis sinensis* is associated with CCA in Korea and China [2]. Other risk factors for CCA are primary sclerosing cholangitis; biliary-duct cysts; hepatolithiasis; and conditions such as hepatitis B infection, hepatitis C infection, obesity, chronic non-alcoholic liver disease, and cirrhosis [3]. The incidence of CCA peaks during the sixth decade of life and shows a slight male predominance. Tumours most frequently occur at the confluence of the hepatic ducts and are classified as hilar CCAs or Klatskin tumours.

The most common physical indications of CCA include symptoms such as jaundice, dyspepsia, and a palpable abdominal mass [1]. Symptoms of CCA depend on whether the tumour mass is located in the intrahepatic area, hilar area, or extrahepatic area. Surgical intervention plays a major role in the treatment of early stage CCA and yields promising results. Patients with unresectable tumours can be treated with palliative procedures such as biliary-enteric bypass or endoscopic or percutaneous stent placement. Palliative surgical bypass is associated with a slightly improved survival rate compared to percutaneous transhepatic biliary drainage. Palliative chemotherapy does not show significant benefits, as survival rates remain low [4].

Common metastatic sites of CCA include the regional lymph nodes and adjacent organs. Distant metastases from CCA are uncommon, but when they do occur, they usually develop in the bone, muscle, brain, and thyroid gland [5-7]. To our knowledge, no other cases of penile metastasis from CCA have been described in the literature. Here, we report the case of a patient who presented with penile metastatic CCA and who was treated in our institution. We aim to provide useful information on this extremely rare condition.

## Case presentation

A 74-year-old Caucasian man consulted us after having undergone penile ultrasonography for pain and increased thickness at the base of the penis after self-examination. The patient presented with a history of hepatitis C-related cirrhosis and intrahepatic CCA, diagnosed 3 years previously, when he underwent right posterior hepatic subsegmentectomy with lymph node dissection. No portal vein invasion had been reported, and all lymph nodes had been negative. The patient had not received adjuvant therapy after surgery. Follow-up examinations had been performed every 3 months for 2 years post-operatively and every 6 months thereafter. The patient had undergone clinical examination, laboratory tests including measurement of tumour markers, abdominal ultrasonography, and radiological investigations including chest and abdominal computed tomography. Twenty-nine months after the primary surgery, follow-up positron emission tomography-computed tomography had not revealed any elevation in the standardised uptake value at any site.

**Figure 1:**
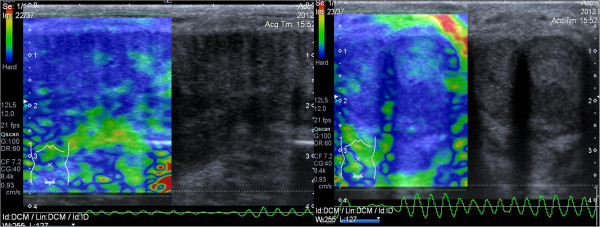
Evidence of large anelastic area in correspondence of the solid lesion located at the level of the corpora cavernosa and the intercavenospongiousus septum with normal elasticity of peripheral tissues.

Penile ultrasound examination, performed with a high-frequency linear probe (12–5 MHz), showed a large solid formation located at the base of the penis with poorly defined edges involving the corpora cavernosa on both sides and the corpus spongiosum of the urethra, without invasion of the tunica albuginea. Elastography showed discrete intralesional vascularity with a high strain ratio of 22. The thickness of the septum between the corpora cavernosa and corpus spongiosum was increased due to calcification on the distal two-thirds of the length (Figure 1).

**Figure 2:**
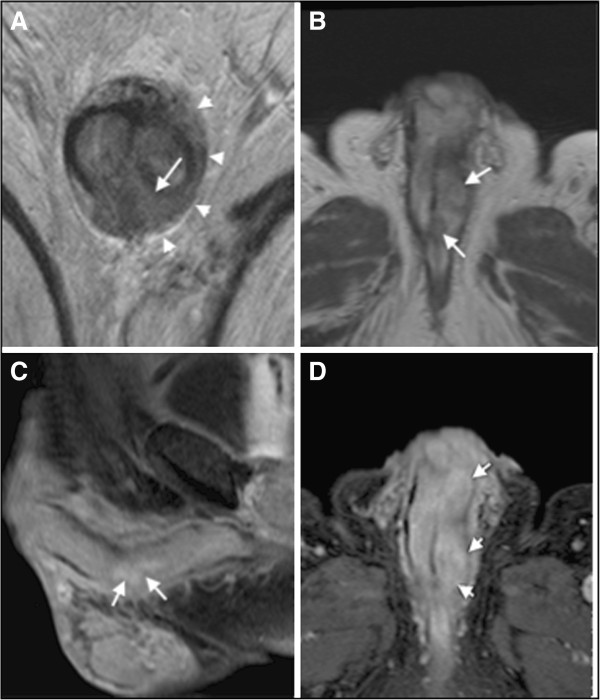
**MRI of the penis. A)** T2 weighted image shows a ipointense tissue sorrounding the left corpus spongiosum (arrow) and arising dorsal and ventral side of tunica albuginea; Buck’s fascia was also involved on the left side (arrowhead). **B)** Unenhanced axial T1-weighted MR image showing hypointense nodular lesions (arrows) on the left corpus spongiosum with irregular margins. **C-D)** Sagittal and axial T1-weighted MR images with fat signal suppression, after Gadolinum injection, show slight enhancement of the corrisponding tissue on **A** and **B**, as we expected on predominant fibrous matrix. None involvement of perivisceral fat was noted.

Magnetic resonance imaging (MRI; Figure 2) showed a mass surrounding the left corpus spongiosum on both dorsal and ventral sides of the tunica albuginea, with involvement of Buck’s fascia. On post-contrast images, the mass showed a slight and tardive enhancement, suggesting a fibrous composition. The urethra was displaced with partial infiltration. MRI was performed without the injection of alprostadil because of the patient’s pain.

**Figure 3:**
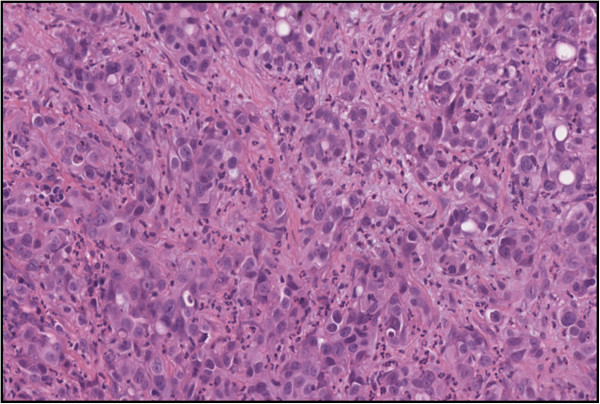
High-power photomicrograph showing metastatic cholangiocarcinoma composed by duct-like structures lined by cuboidal or columnar cells with high pleomorphism, surrounded by dense stromal reaction (magnification 40×).

**Figure 4:**
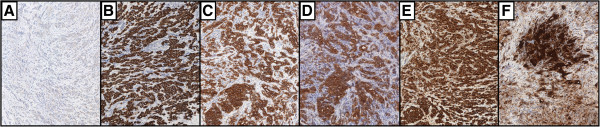
High-power photomicrograph showing immunoreactivity of metastatic (A-F) cholangiocarcinoma; the neoplastic cells resulted negative for Heppar-1 (A) and positive for CK7 (B), CK19 (C), CK 20 (D), EMA, (E) and CEA (F) (magnification 40×).

Discrete intralesional vascularity was also observed. Therefore, we decided to perform a biopsy of the corpora cavernosa on both sides via skin incision at the root of the penis. Histologic analysis revealed a carcinoma harbouring the same histological and immunophenotypical features as the primary hepatic lesion. Histopathologic analysis revealed a malignant epithelial neoplasm composed of cuboidal or columnar cells with pleomorphic nuclei and prominent nucleoli as well as weakly eosinophilic cytoplasm organised in duct-like structures (Figures 3 and 4). The neoplastic cells were positive for CK7, CK 8/18, CK19, CK 20, epithelial membrane antigen (EMA), and carcinoembryonic antigen (CEA) and negative for Heppar-1 and CD56 [8,9].

## Discussion

CCAs form a heterogeneous group of tumours that develop along the biliary tract, arising from different topographic regions of the biliary tree. Depending on their site of origin, they have different features and require different specific treatments. The occurrence of most CCAs is sporadic. Patients with CCA often present with non-specific symptoms such as cachexia, abdominal pain, night sweats, and fatigue. Blood tests that can be used to diagnose CCA are available. CEA and CA19-9 serum levels are often elevated in these patients, but these measurements are not sufficiently sensitive or specific to be used as a general screening tool. However, they may be useful in conjunction with imaging methods, in order to support the diagnosis of a suspected CCA. The common metastatic sites for CCA are the regional lymph nodes (hilar, peripancreatic, and periaortic lymph nodes) and adjacent organs. CCAs are not highly vascularised tumours, making haematogenous metastases uncommon. Haematogenous CCA metastases commonly occur at distant organs such as the lungs, adrenal glands, and bones. Patients with a history of CCA may also develop metastatic disease several years after first presentation. For this reason, the onset of new symptoms or signs requires adequate investigation in these patients, and a possible metastatic occurrence should be considered in order to treat it. In cases of diagnostic doubt, biopsy and histopathology combined with immunophenotypical tests should lead to an accurate diagnosis and prognosis for the patient.

Penile metastasis is always a rare event. Common primary sites are the genitourinary tract (mainly, bladder urothelial cancer and prostate adenocarcinoma) and the lower gastrointestinal tract (especially, rectum and sigmoid colon). Most cases of penile metastasis are metachronous (i.e. prostate, urothelial cancers, etc.).

The most common observed symptoms and signs of metastasis to the penis in order of frequency are priapism (up to 40%, resulting from emboli or thrombosis of dorsal vein, defined as “malignant priapism”), urinary retention, penile nodules, ulcer formation, perineal pain (7–10%), oedema, generalised swelling, broad infiltrative enlargement, dysuria, and haematuria (less than 10%).

The diagnosis in our patient was achieved by ultrasound and MRI that showed the presence of a solid lesion, which was localised in the corpora cavernosa without any involvement of the tunica albuginea and with extensive calcification of the intercavernous and intercavernospongious septa. Moreover, in our case, there were no clinical suspicious or laboratory data (PSA levels, hematuria, etc.), suggestive for malignancies that most frequently metastasize to the penis, such as prostate cancer and urothelial carcinoma.

Several mechanisms by which a tumour can secondarily affect the penis have been described; apart from the retrograde lymphatic route of spread, venous retrograde spread is the most common route, owing to the generous communication between the pelvic venous plexuses and the penile dorsal venous system [7§-9§]. Direct tumour extension has also to be considered a possible mechanism of tumour involvement.

The penile metastasis diagnosis must be established on biopsy, as was performed in this case. To differentiate metastatic lesions from primary penile tumours, an adequate specimen is needed as early as possible. It is important, though, to differentiate primary penile carcinoma from secondary metastatic disease since primary tumours are curable in a high percentage of cases.

The time interval between primary tumour and penile metastasis ranges from 3 months to 5 years and the time interval between diagnosis of penile metastasis and death ranges from 0.25 to 18 months. In our patient, metastasis became evident 3 years after the diagnosis of intrahepatic CCA. Because penile involvement by a secondary tumour usually indicates systemic dissemination, the prognosis is poor. In previously reported series, most of the patients died between 6 months and 1 year after the detection of the penile metastasis [10]. All treatments may be considered palliative, as published clinical cases report improvement of patient symptoms without any important prolongation in survival. Prolonged survival is often possible only when a distal penile lesion or nodule is resectable; limited surgical excision or radiotherapy is usually the most useful modality. Partial or total penectomy will be required with highly invasive neoplasms involving the corporal bodies.

## Conclusions

To date, there is no case of penile or urogenital system metastasis from CCA described in the literature. Therefore, to our knowledge this article represents the first case report of penile metastasis from CCA.

## Consents

Written informed consent was obtained from the patient for publication of this case report. A copy of the written consent is available for review by the Editor-in-Chief of this journal.

## Abbreviations

CCA: Cholangiocarcinoma; CEA: Carcinoembryonic antigen; EMA: Epithelial membrane antigen; MRI: Magnetic resonance imaging.

## Competing interest

None of the contributing authors have any conflict of interest, including specific financial interests or relationships and affiliations relevant to the subject matter or materials discussed in the manuscript.

## Authors’ contributions

ALP, GP, YAS, DD, AF, and GM collected the references and wrote the manuscript. SF, CDC, and NP collected the clinical data. VP and CDR analysed the pathological results. GM, PM, DD, and SF performed echographic and MR examination. AC, CM, and AF initiated the paper, helped to incorporate the clinical data into the manuscript, mentored the writing, and completed the revisions of the manuscript. All authors report no conflicts of interest and approved the final manuscript.

## Pre-publication history

The pre-publication history for this paper can be accessed here:

http://www.biomedcentral.com/1471-230X/13/149/prepub
